# Maternal Vitamin D Deficiency and the Risk of Placental Abruption: A Cross-Sectional Study in a Greek Obstetric Population

**DOI:** 10.3390/clinpract15060102

**Published:** 2025-05-26

**Authors:** Artemisia Kokkinari, Evangelia Antoniou, Eirini Orovou, Maria Dagla, Maria Tzitiridou-Chatzopoulou, Antigoni Sarantaki, Kleanthi Gourounti, Georgios Iatrakis

**Affiliations:** 1 Department of Midwifery, School of Health & Care Sciences, University of West Attica, 12243 Athens, Greece; lilanton@uniwa.gr (E.A.); mariadagla@uniwa.gr (M.D.); esarantaki@uniwa.gr (A.S.); kgourounti@uniwa.gr (K.G.); giatrakis@uniwa.gr (G.I.); 2 Department of Midwifery, School of Health & Care Sciences, University of Western Macedonia, 54636 Kozani, Greece; eorovou@uowm.gr (E.O.); mtzitiridou@uowm.gr (M.T.-C.)

**Keywords:** vitamin D deficiency, placental abruption, pregnancy complications, maternal health, obstetric outcomes, risk factors, prenatal care

## Abstract

Background: Vitamin D deficiency (VDD) during pregnancy has been associated with various obstetric complications, including preeclampsia, gestational diabetes, and premature rupture of membranes. However, its potential link to placental abruption remains underexplored. The aim of this study was to investigate whether low maternal vitamin D levels are associated with an increased risk of placental abruption in pregnancies considered otherwise low-risk. Methods: We conducted a cross-sectional study involving 248 pregnant women who were admitted for delivery at a public hospital in Athens, Greece. Serum levels of 25-hydroxyvitamin D [25(OH)D] were measured upon admission. Levels below 30 ng/mL were classified as insufficient. Although this threshold corresponds to insufficiency according to the Endocrine Society, for the purposes of this study, levels < 30 ng/mL were treated as indicative of vitamin D deficiency in order to capture broader physiological implications. Cases of placental abruption were identified based on obstetric history and clinical documentation at the time of delivery. A Chi-square test was used to assess the association between vitamin D status and placental abruption, and a multivariate logistic regression model was applied to control for potential confounders, including hypertensive disorders of pregnancy, smoking, and preterm birth. The potential role of vitamin D supplementation during pregnancy was also explored as part of the analysis. Results: Our analysis revealed that women with VDD had a significantly higher incidence of placental abruption (*p* < 0.05). In the multivariate model, VDD remained an independent risk factor (adjusted OR: 3.2, 95% CI: 1.1–9.6). Additional risk factors that showed significant associations with placental abruption included pregnancy-induced hypertension and maternal smoking. Conclusions: These findings support the hypothesis that insufficient maternal vitamin D levels may contribute to adverse pregnancy outcomes, including placental abruption. Further prospective studies are warranted to clarify the causal mechanisms and to evaluate whether early detection and correction of vitamin D deficiency could serve as a preventive strategy in prenatal care.

## 1. Introduction

The role of vitamin D in pregnancy has gained increasing attention in recent years, as emerging evidence suggests its involvement in various aspects of maternal and fetal health. The aim of the present study is to investigate the association between maternal vitamin D deficiency (VDD) and the risk of placental abruption in a low-risk obstetric population, while also examining the potential impact of vitamin D supplementation during pregnancy.

VDD is highly prevalent among pregnant women worldwide, including in southern European populations [1]. This high prevalence underscores the global relevance of investigating its clinical implications during pregnancy [1]. Low levels of maternal 25-hydroxyvitamin D [25(OH)D] have been linked to adverse outcomes, such as preeclampsia, gestational diabetes, intrauterine growth restriction, and preterm birth [2,3]. Additionally, maternal VDD has been associated with an increased risk of preeclampsia, a pregnancy complication closely linked to placental dysfunction [4]. These associations have been supported by meta-analytic data, indicating that low maternal vitamin D levels are significantly associated with increased risk of several adverse perinatal outcomes, including small-for-gestational-age (SGA) infants and preeclampsia [5].

The circulating concentration of total serum 25(OH)D is most commonly used to determine vitamin D status in clinical and research settings. However, there is no single universally accepted threshold for defining deficiency or insufficiency. The U.S. Institute of Medicine (IOM) classifies serum 25(OH)D levels below 30 nmol/L (12 ng/mL) as deficient, between 30 and 50 nmol/L (12–20 ng/mL) as insufficient, and levels ≥ 50 nmol/L (20 ng/mL) as sufficient for bone health [6]. In contrast, the Endocrine Society defines VDD as serum 25(OH)D ≤ 50 nmol/L (20 ng/mL), insufficiency as 50–75 nmol/L (20–30 ng/mL), and sufficiency as ≥75 nmol/L (30 ng/mL), based on parathyroid hormone suppression and broader physiological effects [7,8]. In line with these broader physiological considerations, this study adopted a threshold of <30 ng/mL to define suboptimal vitamin D status. For consistency in analysis, this cut-off was used to classify participants as vitamin D deficient. Importantly, all guidelines agree that levels below 25–30 nmol/L (10–12 ng/mL) should be avoided at any age due to the risk of metabolic bone disease and adverse outcomes [9,10].

Recent studies have begun to explore its possible role in placental disorders, including placental abruption—a serious condition associated with significant maternal and neonatal morbidity and mortality. Vitamin D receptors are expressed in the placenta and decidua, suggesting a direct role of vitamin D in implantation and placental development [11]. In addition to serum levels, the role of vitamin D supplementation during pregnancy has also raised questions [12]. While supplementation is commonly recommended in deficient populations, it remains unclear whether it directly reduces the risk of placental complications. Some randomized controlled trials (RCTs) and systematic reviews (SR) have suggested that vitamin D supplementation may reduce the risk of pregnancy-related complications, although findings remain inconclusive [13]. Hence, our study also aims to explore whether supplement use correlates with abruption risk among vitamin-D-deficient women.

Supporting this direction, a stratified randomized field trial conducted in Iran demonstrated that correcting severe VDD through supplementation—raising serum 25(OH)D levels from approximately 11 ng/mL to over 20 ng/mL—was associated with significant reductions in adverse pregnancy outcomes [14]. These findings suggest that achieving even modest improvements in vitamin D status may have clinical relevance, particularly among populations with high rates of deficiency.

Some observational studies have suggested that insufficient vitamin D levels may contribute to the pathophysiology of placental abruption through impaired placental implantation, increased inflammation, or altered angiogenesis [12,15]. It is important to note that our study defined VDD using a threshold of <30 ng/mL, in contrast to the stricter < 20 ng/mL definition often cited. This choice was made to encompass both deficient and insufficient levels that may be clinically relevant in pregnancy. Longitudinal studies have also suggested that persistent vitamin D deficiency throughout pregnancy may increase the risk of placental complications, such as preeclampsia and possibly placental abruption [16]. Several studies have suggested a link between VDD and placental disorders, including placental abruption. For instance, studies have shown that low vitamin D levels may contribute to placental dysfunction through various mechanisms, such as altered placental vasculature or increased inflammation [12,15]. Additionally, maternal vitamin D status has been shown to influence placental gene expression and vascular remodeling, which may further contribute to complications, such as placental abruption [17]. However, the evidence remains inconsistent, with some studies finding no statistically significant relationship between maternal vitamin D status and placental abruption [12,18].

This association remains controversial, as current evidence is considered limited and inconclusive due to methodological heterogeneity, lack of adjustment for confounding factors, or small sample sizes [12,18]. For example, Vestergaard et al. [12] emphasize the need for larger prospective trials to better clarify this potential relationship.

Given these conflicting findings, further research is warranted. Our study contributes to this field by examining the association in a well-defined Greek obstetric cohort using both univariate and multivariate analyses to control for potential confounders.

Emerging research has increasingly explored the potential link between maternal VDD and placental pathologies, such as abruption. Vitamin D appears to play a regulatory role in placental inflammation, vascular remodeling, and trophoblast invasion—critical processes for normal placental development. Liu et al. [19] demonstrated that vitamin D can modulate immune responses in the placenta by suppressing pro-inflammatory cytokines, suggesting a protective mechanism against inflammatory-mediated placental damage. Similarly, Chen et al. [20] showed in an animal model that gestational VDD led to placental insufficiency and fetal growth restriction, primarily through enhanced placental inflammation. Clinical research further supports this hypothesis; Vestergaard et al. [21] reported that vitamin D insufficiency during pregnancy was associated with altered placental vitamin D metabolism and increased risk of adverse outcomes. In a retrospective cohort study, Yates et al. [22] observed that low maternal vitamin D levels in early pregnancy were associated with a higher incidence of placental abruption, providing initial clinical evidence for this association. These findings collectively suggest a potential pathophysiological link between VDD and the risk of placental abruption.

## 2. Materials and Methods

This cross-sectional observational study was conducted at the Obstetrics and Gynecology Department of Tzaneio General Hospital in Piraeus, Greece, from September 2019 to January 2022. The aim was to investigate a possible association between maternal VDD and the occurrence of placental abruption in a low-risk pregnant population.

### 2.1. Inclusion and Exclusion Criteria

Eligible participants were pregnant Greek women aged 18 years and older who gave birth at ≥37 weeks of gestation to a live singleton neonate at the study hospital. Women were excluded if they had a known history of conditions potentially affecting vitamin D metabolism, such as autoimmune, endocrine (e.g., thyroid, adrenal or parathyroid), hepatic, renal, or malabsorptive disorders. Further exclusion criteria included use of medications that may influence vitamin D levels (e.g., corticosteroids, antiepileptics, antifungals, or antituberculosis drugs) and high-dose vitamin D supplementation (>800 IU/day). Pregnancies complicated by multiple gestation, stillbirth, or major fetal anomalies were also excluded.

### 2.2. Data Collection

A total of 248 women were included. Demographic data, obstetric history, and lifestyle information (smoking, physical activity, vitamin D intake, etc.) were collected via structured questionnaires administered postpartum. Clinical information was extracted from medical records.

Blood samples were obtained from all participants during routine pre-delivery laboratory testing. Maternal serum 25-hydroxyvitamin D [25(OH)D] levels were measured on the day of delivery using a validated ELISA assay. VDD was defined as 25(OH)D levels < 30 ng/mL, according to the guidelines of the Endocrine Society.

Placental abruption was diagnosed clinically and confirmed by obstetricians based on standardized criteria, including vaginal bleeding, uterine tenderness, and evidence of retroplacental clot or abruption on placental inspection.

### 2.3. Vitamin D Supplementation Status

Information regarding prenatal vitamin D supplementation was recorded. Some women received daily supplementation of vitamin D (400–800 IU), based on the discretion of their treating physicians, in accordance with Greek national guidelines. The study investigators did not intervene in clinical decisions but documented whether or not supplementation was used.

### 2.4. Statistical Analysis

Participants were categorized according to their vitamin D status (VDD vs. adequate levels) and further stratified by use of vitamin D supplementation. The incidence of placental abruption was calculated and compared between groups.

Univariate analyses were performed using the Chi-square test to identify potential associations between maternal VDD, vitamin D supplementation, and placental abruption. Multivariate logistic regression was subsequently applied to assess whether maternal VDD was an independent risk factor for placental abruption, adjusting for potential confounders, such as hypertensive disorders of pregnancy, maternal smoking, and preterm birth. Odds ratios (OR) with 95% confidence intervals (CI) were reported. A *p*-value < 0.05 was considered statistically significant.

All analyses were performed using IBM SPSS Statistics version 26.0 software and Microsoft EXCEL (v. 2010; Microsoft corp Redmond).

### 2.5. Ethical Considerations

This study was conducted in accordance with the ethical standards of the Declaration of Helsinki and was approved by the Ethics Committee of the Tzaneio General Hospital of Piraeus (Approval No: 6/6 June 2019; Application No: 7380/27 May 2019). All participants were fully informed about the purpose and procedures of the study and provided written informed consent prior to enrollment.

Participants were assured of the confidentiality of their personal data, which were anonymized and stored securely. They were also informed that their participation was voluntary and that they could withdraw from the study at any time without affecting the medical care they would receive.

Biological samples (maternal and cord blood) were collected as part of routine clinical care and used solely for the purposes of vitamin D analysis, as outlined in the study protocol. No additional interventions or risks were introduced to mother or neonate. The study adhered to all relevant national and institutional guidelines for biomedical research involving human subjects.

### 2.6. Literature Review Strategy

The literature review that informed the background and justification for this study was conducted between March and July 2023. Although the clinical phase of the study was completed in January 2022, the literature review was conducted between March and July 2023 to incorporate the most up-to-date evidence available at the time of manuscript preparation. A systematic search was performed across multiple databases, including PubMed, Scopus, and Google Scholar, to identify peer-reviewed studies relevant to maternal vitamin D status and placental complications, particularly placental abruption. The search strategy employed a combination of keywords and Boolean operators: (“vitamin D” OR “25-hydroxyvitamin D” OR “hypovitaminosis D”) AND (“placental abruption” OR “placental disorders” OR “pregnancy outcomes”).

Inclusion criteria for the references were as follows: (1) original research articles, (2) systematic reviews or meta-analyses, (3) human studies, and (4) English-language publications. Priority was given to studies published after 2020, although some earlier high-quality and widely cited studies were retained for context and historical relevance. Articles were excluded if they involved animal models only, were non-English, or focused exclusively on unrelated outcomes (e.g., cancer risk, autoimmune disease). Reference lists of key papers were also hand-searched to identify additional relevant sources.

This structured search approach ensured a comprehensive and current representation of the literature, while maintaining relevance to the research question.

## 3. Results

A total of 248 pregnant women were included in this cross-sectional study. The mean age of participants was 30.5 ± 5.2 years, with a mean gestational age of 38.6 ± 2.1 weeks. The prevalence of VDD among the study population was 42%, with 104 women having serum 25-hydroxyvitamin D levels below 30 ng/mL (Table 1a).

Among the 248 women, 15 cases of placental abruption were identified, corresponding to a 6.1% incidence rate. Women with VDD had a significantly higher incidence of placental abruption compared to women with adequate vitamin D levels (9.6% vs. 3.4%, *p* < 0.05) (Table 1a).

In the multivariate logistic regression analysis, adjusting for potential confounders, including hypertensive disorders of pregnancy, smoking, and preterm birth, VDD remained an independent risk factor for placental abruption (adjusted OR: 3.2, 95% CI: 1.1–9.6; *p* = 0.03) (Table 2a). The association between VDD and placental abruption was also observed in women who did not use vitamin D supplementation during pregnancy. However, no significant association was found between the use of vitamin D supplementation and the risk of placental abruption (*p* = 0.51, Table 2a). Other significant risk factors for placental abruption included pregnancy-induced hypertension (OR: 2.9, 95% CI: 1.4–6.0) and maternal smoking (OR: 3.1, 95% CI: 1.2–7.8), as well as hypertension (OR: 2.5, 95% CI: 1.2–5.0, *p* = 0.02) (Table 2a).

To explore whether the risk was particularly elevated in more severe deficiency, we conducted a secondary analysis using the Endocrine Society’s cut-off definitions. Participants were categorized into three groups: deficiency (<20 ng/mL), insufficiency (20–30 ng/mL), and sufficiency (>30 ng/mL). Women with VDD showed the highest incidence of placental abruption (13.3%), followed by the insufficient group (4.5%) and the sufficient group (3.4%) (Table 1b). In the adjusted logistic regression model, vitamin D deficiency (<20 ng/mL) remained significantly associated with placental abruption (adjusted OR: 4.1, 95% CI: 1.4–12.2, *p* = 0.009), whereas insufficiency did not (*p* = 0.63) (Table 2b).

**Figure 1:**
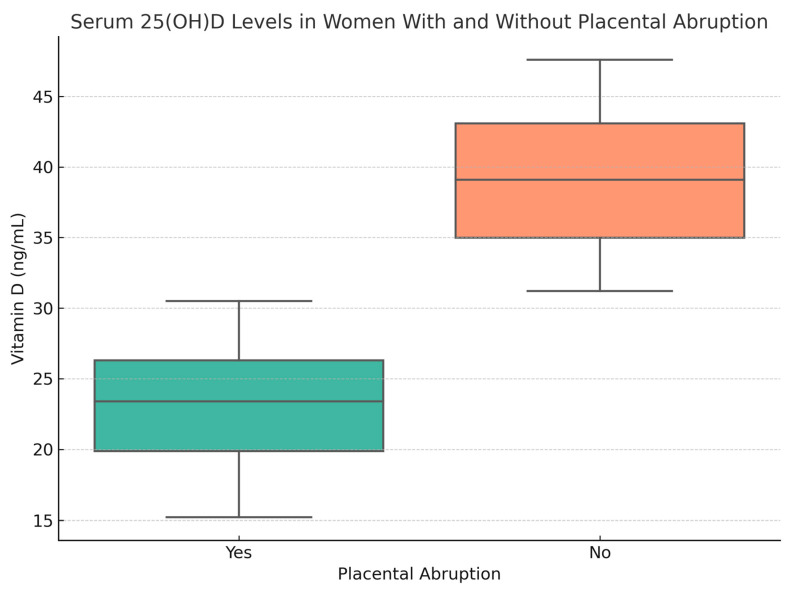
Boxplot showing the distribution of maternal serum 25(OH)D levels among women with and without placental abruption. Lower median values were observed in the abruption group, consistent with the identified statistical association.

A visual representation of the distribution of serum 25(OH)D concentrations according to the presence or absence of placental abruption is provided in Figure 1. The figure illustrates the lower median and interquartile range of vitamin D levels among women who experienced placental abruption, supporting the observed statistical association. This graphical depiction highlights the inverse relationship between serum 25(OH)D and abruption risk and complements the regression findings by emphasizing the clustering of abruption cases at lower vitamin D levels (Figure 1).

As shown in Table 3, although a higher proportion of women with placental abruption had not received vitamin D supplementation (80% vs. 83% in those without abruption), the difference was not statistically significant (*p* = 0.65). This finding aligns with the multivariate analysis (Table 2a), where vitamin D supplementation was also not a significant predictor (*p* = 0.51).

## 4. Discussion

Our study demonstrates a significant association between maternal vitamin D deficiency (VDD) and an increased risk of placental abruption in a low-risk obstetric population. This finding aligns with previous research suggesting that inadequate vitamin D levels during pregnancy may contribute to the pathophysiology of placental dysfunction through mechanisms like impaired placental implantation, altered angiogenesis, and increased inflammation [12,15]. However, it is important to note that as our study is cross-sectional in nature, it cannot establish causality but rather indicates an association between maternal vitamin D deficiency and the increased risk of placental abruption. Longitudinal studies are needed to better understand the causal relationship between vitamin D levels and placental complications.

Interestingly, we observed that vitamin D supplementation did not appear to significantly reduce the risk of placental abruption in women with VDD, which raises questions about the efficacy of supplementation in preventing this specific complication. This is in partial contrast to the findings by [14], who reported that vitamin D supplementation significantly reduced adverse pregnancy outcomes. However, unlike our study, their trial focused on broader pregnancy complications, not placental abruption specifically, and involved earlier intervention during gestation. These differences in study design may explain the discrepancy. While supplementation is commonly recommended in vitamin-D-deficient populations, the role of supplementation in reducing placental complications remains unclear [12]. Nonetheless, a stratified randomized field trial in Iran demonstrated that increasing serum 25(OH)D levels from approximately 11 ng/mL to over 20 ng/mL through supplementation significantly reduced adverse pregnancy outcomes, highlighting the potential effectiveness of even modest improvements in vitamin D status [14]. This discrepancy between the observed association and the lack of a significant protective effect of supplementation warrants further investigation. It is possible that the dose of supplementation, the timing of administration during pregnancy, or individual variations in vitamin D metabolism may influence its effectiveness.

Previous studies have shown inconsistent results regarding the relationship between VDD and placental abruption. For instance, Vestergaard et al. [12] observed a positive association between low maternal vitamin D levels and placental abruption, which supports our findings. However, their study population included women with pre-existing comorbidities, which may have amplified the observed risk. Our focus on a low-risk population highlights that the association persists even in healthier cohorts, strengthening the argument for a more generalizable link. Other studies, however, have reported a positive correlation between low vitamin D levels and placental disorders, whereas others have found no statistically significant relationship [12,18]. These inconsistencies may be attributed to methodological differences, such as varying sample sizes, different definitions of vitamin D deficiency, and unaccounted confounders. For example, some studies might not have fully adjusted for other potential risk factors, such as hypertension or smoking, which are also known to affect placental function. Our study attempts to address these limitations by adjusting for key confounders, including hypertensive disorders of pregnancy, smoking, and preterm birth.

Moreover, stratified analysis using clinically relevant serum 25(OH)D categories (<20 ng/mL, 20–30 ng/mL, and >30 ng/mL) revealed that women with VDD (<20 ng/mL) had a significantly higher risk of placental abruption compared to those with sufficient levels. These categories were defined based on the Endocrine Society’s clinical guidelines, which recommend maintaining serum 25(OH)D levels above 30 ng/mL for optimal health during pregnancy [7]. This supports previous findings indicating a threshold effect around this value. Alternative cut-off points (e.g., 25, 20, 15 ng/mL) were also tested, with <20 ng/mL consistently demonstrating the strongest association. These results highlight the importance of precise categorization when evaluating the impact of vitamin D on adverse pregnancy outcomes. It should be noted that although the threshold of <20 ng/mL is widely used to define VDD, our study employed a broader definition of suboptimal vitamin D status, including both deficient (<20 ng/mL) and insufficient (20–30 ng/mL) levels, in line with the Endocrine Society guidelines [7]. This decision was made to capture a wider range of physiologically relevant effects and to better reflect the potential impact of borderline insufficiency on placental health. As such, participants with serum 25(OH)D concentrations below 30 ng/mL were categorized as vitamin D deficient for analytical purposes.

Our results are in line with a growing body of literature that suggests a biological plausibility for the association between maternal vitamin D deficiency and placental abruption. Liu et al. [19] highlighted the role of vitamin D in downregulating inflammatory pathways within the placenta, which may be critical in preventing placental ischemia or detachment. Experimental findings by Chen et al. [20] further support this by showing that vitamin-D-deficient pregnancies exhibited increased placental inflammation and reduced placental function, leading to fetal growth restriction. These mechanistic insights complement our clinical observations and may explain the pathway through which VDD contributes to placental abruption. The alignment between clinical and experimental evidence reinforces the plausibility of our findings. Moreover, Vestergaard et al. [21] found that pregnant women with low vitamin D levels had altered placental vitamin D metabolism and a higher risk of obstetric complications. Importantly, Yates et al. [22] provided clinical evidence that maternal vitamin D deficiency in early gestation was associated with a higher incidence of placental abruption in a large cohort. Taken together, these data support the hypothesis that adequate maternal vitamin D levels may be essential for maintaining placental integrity and preventing abruptions. Recent studies further reinforce the link between vitamin D deficiency and placental dysfunction relevant to placental abruption. Chen et al. [20] demonstrated that gestational vitamin D deficiency induces inflammatory responses in the placenta, leading to placental insufficiency and fetal growth restriction. Similarly, Boskabadi et al. [23] found that neonates born to mothers with vitamin D deficiency had significantly lower Apgar scores and higher rates of perinatal complications, suggesting compromised intrauterine environments potentially associated with placental impairment. While their focus was neonatal outcomes, the observed adverse effects likely stem from placental dysfunction, aligning with our hypothesis that VDD-related placental changes contribute to abruption and other perinatal complications. Additionally, a recent systematic review by Gerovasili et al. [24] emphasized that vitamin D deficiency may impair placental angiogenesis and increase inflammatory mediators, contributing to suboptimal placental development. These findings highlight the importance of adequate maternal vitamin D status in maintaining placental health and preventing adverse outcomes, such as abruption.

Moreover, our findings are consistent with the hypothesis that other risk factors, such as pregnancy-induced hypertension and maternal smoking, may interact with VDD to increase the risk of placental abruption. These factors have been previously reported to contribute to placental dysfunction and should be considered when managing pregnant women with VDD. Pregnancy-induced hypertension, for example, has been shown to cause placental ischemia and impaired blood flow, which may exacerbate the risk of placental abruption [25]. Similarly, maternal smoking has been associated with endothelial dysfunction and altered placental blood flow, both of which may contribute to placental insufficiency [26]. The interaction between these factors highlights the complexity of placental abruption and suggests that vitamin D deficiency is just one piece of the puzzle.

The prevalence of VDD observed in our study (42%) is consistent with findings from other populations, particularly in southern Europe, where lower levels of vitamin D during pregnancy are common [1]. This suggests that VDD is a widespread issue that warrants attention in prenatal care. Ensuring adequate vitamin D levels during pregnancy may be crucial for maternal and fetal health, especially in regions with high rates of deficiency. However, the best approach to correcting vitamin D deficiency, particularly in relation to placental outcomes, remains unclear and requires further research.

Furthermore, considering that the fetus is entirely dependent on the placental transfer of maternal 25-hydroxyvitamin D, any disruption in placental function, such as placental abruption, could potentially alter fetal cord blood 25-hydroxyvitamin D concentrations. This aspect introduces an additional layer of complexity, as it suggests a bidirectional relationship; while maternal VDD may contribute to the risk of placental abruption, the occurrence of abruption itself could also impact the assessment of fetal vitamin D status at birth. Future studies measuring cord blood levels in relation to placental pathology may help elucidate this potential interaction.

Our study has several strengths, including its focus on a well-defined, low-risk obstetric cohort and its use of both univariate and multivariate analyses to control for potential confounders. Nevertheless, there are some limitations to consider. First, the cross-sectional design limits our ability to draw causal conclusions. Longitudinal studies that track vitamin D levels throughout pregnancy and their relationship with placental outcomes would provide more robust evidence. Additionally, while we adjusted for several important confounders, residual confounding due to factors not accounted for in the analysis cannot be ruled out. Finally, the assessment of vitamin D levels was based on a single measurement at the time of delivery, which may not accurately reflect maternal vitamin D status throughout the entire pregnancy.

In conclusion, our study suggests that maternal VDD is associated with an increased risk of placental abruption in a low-risk obstetric population. However, the role of vitamin D supplementation in preventing this complication remains unclear, and further research is needed to clarify the potential benefits of supplementation and to identify the optimal approach to correcting VDD during pregnancy. In light of the complexity of placental abruption, future studies should consider the interactions between vitamin D levels and other risk factors, such as hypertension and smoking, to better understand the underlying mechanisms and improve prenatal care strategies.

## 5. Conclusions

This study provides evidence that maternal VDD is an independent risk factor for placental abruption. Moreover, it should be considered that placental abruption itself may influence fetal cord blood vitamin D concentrations, adding complexity to the interpretation of neonatal vitamin D status in such cases. However, the potential role of vitamin D supplementation in reducing this risk remains inconclusive. Given the current limitations and inconsistencies in the literature, further prospective studies are needed to clarify the causal mechanisms between VDD and placental disorders. Additionally, larger trials are required to assess whether early detection and correction of VDD could be an effective preventive strategy in prenatal care.

Our findings also underscore the importance of utilizing clinically meaningful serum 25(OH)D thresholds when assessing the risk of placental complications. While we used the 30 ng/mL threshold to define insufficiency based on Endocrine Society guidelines, our stratified analysis provided a more detailed risk gradient across clinically relevant subcategories.

Stratified analysis revealed that vitamin D levels below 20 ng/mL were most strongly associated with adverse outcomes, suggesting that future preventive strategies should take into account this critical threshold.

## Figures and Tables

**Table 1:** (**a**) Study characteristics and distribution of participants. (**b**) Study characteristics and distribution by serum 25(OH)D categories.

(a)				
Characteristics	Total (*n* = 248)	<30 ng/mL (*n* = 104)	≥30 ng/mL (*n* = 144)	*p*-Value
Age (Years)	30.5 ± 5.2	30.2 ± 5.1	30.8 ± 5.3	0.21
Gestational Age (Weeks)	38.6 ± 2.1	38.4 ± 2.2	38.7 ± 2.0	0.18
Preeclampsia	8 (3.2%)	4 (3.8%)	4 (2.8%)	0.56
Gestational Diabetes	9 (3.6%)	5 (4.8%)	4 (2.8%)	0.35
Preterm Birth	12 (4.8%)	6 (5.8%)	6 (4.2%)	0.61
Placental Abruption	15 (6.1%)	10 (9.6%)	5 (3.4%)	0.03 *
(**b**)				
**Characteristic**	**<20 ng/mL (*n* = 11)**	**20–30 ng/mL (*n* = 93)**	**>30 ng/mL (*n* = 144)**	***p*-Value**
Number of Participants	11	93	144	
Age (Years)	30.0 ± 5.1	30.2 ± 5.1	30.8 ± 5.3	0.21
Gestational Age (Weeks)	38.3 ± 2.3	38.4 ± 2.2	38.7 ± 2.0	0.18
Preeclampsia	1 (9.1%)	3 (3.2%)	4 (2.8%)	0.56
Gestational Diabetes	1 (9.1%)	4 (4.3%)	4 (2.8%)	0.35
Preterm Birth	1 (9.1%)	5 (5.4%)	6 (4.2%)	0.61
Placental Abruption	2 (18.2%)	8 (8.6%)	5 (3.4%)	0.01 *

* Statistically significant at *p* < 0.05.

**Table 2:** (**a**) Multivariate logistic regression analysis for risk of placental abruption. (**b**) Multivariate logistic regression analysis with redefined deficiency.

(a)		
Factors	OR (95% CI)	*p*-Value
Vitamin D Deficiency (VDD)	3.2 (1.1–9.6)	0.03 *
Vitamin D supplementation (Yes/No)	0.8 (0.4–1.6)	0.51
Preeclampsia	2.9 (1.4–6.0)	0.01 *
Hypertension (Yes/No)	2.5 (1.2–5.0)	1.2–5.0
Maternal Smoking	3.1 (1.2–7.8)	0.02 *
Preterm Birth	1.8 (0.8–4.2)	0.15
(**b**)		
**Factor**	**OR (95% CI)**	***p*-Value**
Vitamin D Deficiency (<20)	4.1 (1.4–12.2)	0.009 **
Insufficiency (20–30)	1.4 (0.3–5.9)	0.63
Preeclampsia	2.9 (1.4–6.0)	0.01 *
Maternal Smoking	3.0 (1.2–7.6)	0.02 *
Preterm Birth	1.7 (0.7–4.1)	0.19

* Statistically significant at *p* < 0.05; ** *p* < 0.01.

**Table 3:** The role of vitamin D supplementation in placental abruption.

Vitamin D Supplementation Use	Placental Abruption (*n* = 15)	No Placental Abruption (*n* = 233)	*p*-Value
Yes	3 (20%)	40 (17%)	0.65
No	12 (80%)	193 (83%)	0.45

## Data Availability

The data are not publicly available due to the Principle of Personal Data protection regulations but can be obtained upon a reasonable request to the corresponding author.
